# Synthesis and Performance of Deep-Red Phosphorescent Iridium Complexes with Pyrone as an Auxiliary Ligand

**DOI:** 10.3390/molecules29133183

**Published:** 2024-07-03

**Authors:** Wen Jiang, Wenming Hou, Caixian Yan, Zhifeng Nie, Qiaowen Chang, Xiangguang Li, Weiping Liu

**Affiliations:** 1 Kunming Institute of Precious Metals, Yunnan Precious Metals Laboratory Co., Ltd., Kunming 650106, China; jiangwen0730@126.com (W.J.); ycx19860706@163.com (C.Y.); liuweiping0917@126.com (W.L.); 2 Yunnan Key Laboratory of Metal-Organic Molecular Materials and Device, School of Chemistry and Chemical Engineering, Kunming University, Kunming 650214, China; niezf123@kmu.edu.cn; 3 Sino-Platinum Metals Chemical (Yunnan) Co., Ltd., Kunming 650503, China; hwm@ipm.com.cn

**Keywords:** pyrone, deep red, iridium complex, phosphorescent organic light-emitting diode (PhOLED)

## Abstract

Two bis-cyclometalated heteroleptic iridium complexes incorporating 1-phenylisoquinoline (piq) as the main cyclometalating ligand and 3-hydroxy-2-methyl-4-pyrone (ma) or 2-ethyl-3-hydroxy-4H-pyran-4-one (ema) as the auxiliary ligand, namely Ir(piq)_2_(ma) (Ir-1) and Ir(piq)_2_(ema) (Ir-2), were developed and applied as deep-red phosphors in organic light-emitting diodes (OLEDs). The two auxiliary ligands had similar influences on the photophysical, electrochemical, and electroluminescent properties of the iridium complexes. Ir(piq)_2_(ma) (Ir-1) showed better luminescence performance in a simple phosphorescent OLED compared to the traditional red iridium complex Ir(piq)_2_(acac) and exhibited a current efficiency of 9.39 cd A^−1^ (EQE of 12.09%). In contrast, Ir(piq)_2_(ema) exhibited an efficiency of 8.6 cd A^−1^ (EQE of 10.19%).

## 1. Introduction

Electroluminescence (EL) directly converts electrical energy into light in response to an electric current or under the action of an electric field. In the 1960s, Pope et al. [1] applied a high-voltage direct current to single-crystal anthracene and observed EL in this organic semiconductor, but due to the high driving voltage of the device and low luminosity, it did not receive much attention at the time. About 30 years later, scholars discovered phosphorescent EL and prepared the first phosphorescent organic light-emitting diode (PhOLED) [2]. Due to the strong spin–orbit coupling of transition metal atoms, the PhOLED’s theoretical internal quantum efficiency reached 100% as a light-emitting material, thus overcoming the theoretical quantum efficiency limit of 25% for the first time. This laid the foundation for further commercialization efforts, allowing EL materials to be rapidly developed.

Luminescent materials based on transition metal complexes have high thermal stability, high quantum efficiency, and easily adjustable luminescent colors. Transition metals (mainly Ru, Os, Pt, and Ir) have d 6 and d 8 electron arrangements in their valence shells to provide empty orbitals and often include ligands to provide a lone pair of electrons. Among heavy-metal complexes, metallacyclic Ir(III) complexes have the best comprehensive performance and are the most promising EL materials. This is mainly due to the following: (1) the atomic number of Ir is large, which allows the complexes to produce strong spin–orbit coupling; (2) the d-orbital splitting of iridium ions is large when forming complexes, which keeps its energy level away from the metal-to-ligand charge transfer (MLCT) energy level of the complexes, thereby avoiding MLCT to the metal’s d orbitals; and (3) the trivalent ion Ir can form stable neutral molecules with ligands, which is beneficial to device fabrication using evaporation or solution processing. Depending on the desired luminescence color, metallacyclic Ir(III) complexes can achieve color tunability from blue to deep red, while maintaining their thermal and electrochemical stability [3,4,5,6,7].

Great success has been achieved in developing green, yellow, and orange Ir(III) complexes [8,9,10], but the development of efficient and stable phosphorescent emitters from saturated red to deep red is still an unsolved problem. This is mainly due to the small energy level difference between the ground state and the lowest triple excited state of red-emitting iridium complexes, which enhances non-radiative leaps and reduces quantum efficiency. To obtain the maximum red shift in the luminescence wavelength, ligands with large π-conjugated systems, including benzothiophenes, benzothiazoles, quinolones, and isoquinolines, are required. However, increasing the length of conjugated systems strengthens interactions between complex molecules, and the large conjugated planar molecules tend to undergo π–π stacking and aggregate, which can lead to serious triplet–triplet annihilation (TTA) and triplet–polaron annihilation (TPA). In addition, the lower triplet energy level also increases self-quenching, and the red emission peak is often broad, making it difficult to achieve red light with high color purity. These constraints have limited the development and application of red phosphorescent materials.

To obtain pure-red phosphorescent emitters, most research works have explored different kinds of homoleptic (Ir(C^N)_3_) [11,12] and heteroleptic (Ir(C^N)_2_(LX)) iridium complexes. In the heteroligand iridium(III) complex Ir(C^N)_2_(LX), the auxiliary ligand LX modulates the optical properties of the central metal Ir(III) ion through its coordination field strength and electronic effects. The introduction of different auxiliary ligands also affects the luminescence of molecular Ir phosphorescent materials. Changing the auxiliary ligand affects not only the photophysical performance of these materials, such as their emission spectrum and quantum yield, but also their thermal stability, film formation, and carrier transport. Among iridium photomolecules, common auxiliary ligands include O^O [13,14,15], N^O [16,17,18,19], S^S [20,21,22], N^N [23,24,25], and P^P [26], of which the most common auxiliary ligand is O^O. The β-diketone O^O double-dentate ligand can coordinate with Ir(III) and form stable octahedral complexes and is the most common auxiliary ligand. It modulates the phosphorescence emission of Ir phosphorescent molecular materials mainly via the triplet energy level [27]. In doped devices, a β-diketone with a higher triplet energy level must be selected to improve the spectral characteristics of the material and its color purity. It can also significantly reduce the sublimation temperature, improve the sublimation effect, and further improve the maximum external quantum efficiency (EQE) [28]. The most common β-diketone auxiliary ligand is acetylacetone (acac). Forrest et al. [29] reported (Btp)2Ir(acac) as the first red Ir complex and doped it into CBP to fabricate a phosphorescent device with an emission wavelength of 616 nm, a maximum EQE of 6.5%, and CIE chromaticity coordinates of (0.68, 0.32). These were close to those of standard saturated red light, but the turn-on voltage was high, and self-quenching at a high current density was intensified, resulting in a significant efficiency roll-off. Liu et al. [30] also reported a series of red phosphorescent Ir complexes with 1-phenylisoquinoline and acetylacetone as the metallacyclic ligand and the auxiliary ligand, respectively. At a current density of 20 mA cm^−2^, the EQE of Ir(piq)_2_(acac) reached 8.46%, with CIE chromaticity coordinates of (0.68, 0.32). More studies have been conducted to increase the length of π-conjugated systems by introducing different substituents using 1-phenylisoquinoline as the host to red-shift the spectrum and optimize the luminescent performance [31,32]. However, phosphorescent iridium complexes are difficult to synthesize, which has prevented their mass production and commercialization.

Most previously reported iridium phosphors with 1-phenylisoquinoline as the cyclometalating ligand show yellow-to-orange phosphorescence, with maximum wavelengths below 630 nm. This study is the first to propose a novel Ir phosphorescent material in which pyrone was selected as the auxiliary ligand without changing the metallacyclic ligand 1-phenylisoquinoline. The luminescent performance of the resulting red phosphorescent iridium complexes was improved without increasing the number of synthesis steps. Pyrone is a diketone compound with a unique structure similar to that of β-diketones and can strongly coordinate with transition metals to form stable complexes [33,34]. The delocalized π electrons in pyrone can chelate with metallacyclic ligands to regulate the luminescent performance, but pyrone has not been reported as a ligand for Ir. In this study, Ir phosphorescent molecular materials were synthesized by using 1-phenylisoquinoline as a metallacyclic ligand and two pyrones as auxiliary ligands, and their photophysical, electrochemical, and EL performances were systematically investigated. Combined with theoretical calculations, the internal relationship between structural characteristics and luminescent performance was explored to provide a reference for the development of red Ir phosphorescent molecular materials with higher efficiency and easier synthesis protocols.

## 2. Results and Discussion

### 2.1. Thermal Stability and Crystalline Structures

Complexes Ir-1 and Ir-2 exhibited thermal decomposition temperatures (Td, corresponding to 5% weight loss) of 308 °C and 320 °C, respectively. 

**Figure 1:**
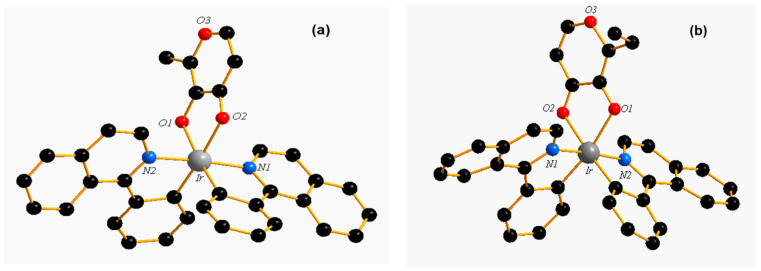
Crystal structures of (**a**) Ir-1 and (**b**) Ir-2.

The crystal structures of Ir-1 and Ir-2 are shown in Figure 1, which shows that the molecular configurations of the crystals of the two Ir complexes were similar. Specifically, the ligands in both crystals formed a slightly distorted octahedral-coordinated structure with a central Ir atom. The C and N atoms involved in the coordination of the main ligand formed a coordination angle of about 80° with the central Ir atom. The two O atoms in the pyrone auxiliary ligand formed a five-membered ring chelate with the central Ir atom, with a coordination angle of about 78°. The partial crystal data of the two crystals are shown in Table 1 and Table 2, in which the bond lengths of Ir–C, Ir–N, and Ir–O in Ir-1 were equal, but there was a small difference in the bond lengths of the Ir atom and other atoms in Ir-2. The two bond angles for C–Ir–O, N–Ir–O, and C–Ir–N in Ir-1 were equal, and the difference between the two bond angles for C–Ir–O and N–Ir–O in Ir-2 was about 6°. The difference between the two bond angles for C–Ir–N was much larger, reaching 16–18°. The symmetry of Ir-1 was better than that of Ir-2, while Ir-2 was distorted because the auxiliary ligand of Ir-2 had more –CH_2_– groups than Ir-1, which caused stronger steric hindrance of auxiliary ligands, which decreased the symmetry and increased the distortion. A higher symmetry may result in the improvement of its photophysical properties.

### 2.2. Photophysical Properties

**Scheme 1:**
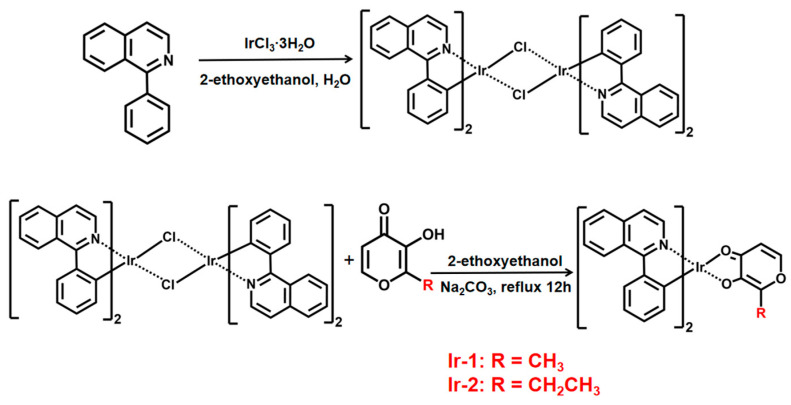
Synthetic routes of complexes Ir-1 and Ir-2.

**Figure 2:**
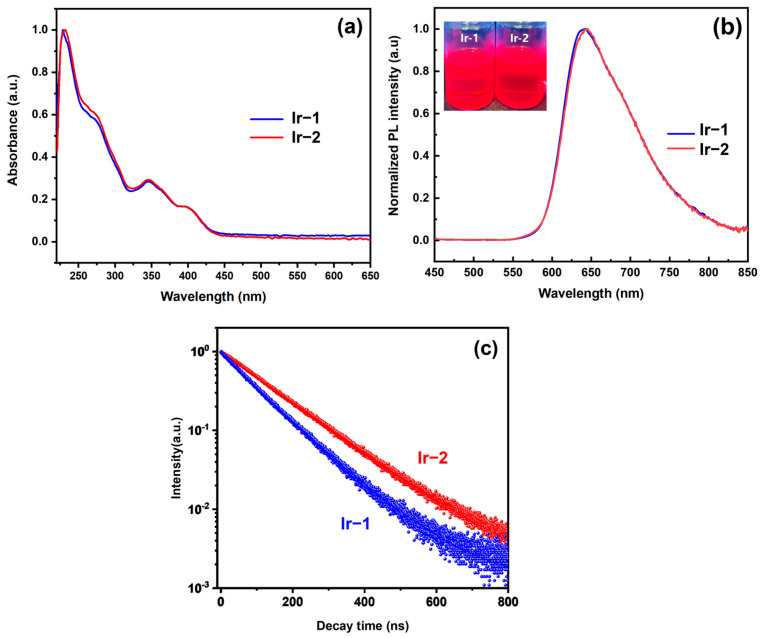
(**a**) UV−VIS absorption spectra of Ir-1 and Ir-2. (**b**) PL spectra of Ir-1 and Ir-2 at 398 nm excitation wavelength. (**c**) Transient PL decay curves.

The synthetic routes of (piq)_2_Ir_2_Cl_2_(piq)_2_ and the Ir(III) complexes Ir-1 and Ir-2 are illustrated in Scheme 1. Photophysical data of Ir-1 and Ir-2 are shown in Table 3. Both Ir-1 and Ir-2 could meet the thermal stability requirements of OLED devices for luminous materials. The UV–VIS absorption and PL spectra of complexes Ir-1 and Ir-2 were measured in dilute CH_2_Cl_2_ at room temperature and are depicted in Figure 2a,b. The complexes Ir-1 and Ir-2 exhibited similar absorption spectra, which consisted of π−π*-type and metal-to-ligand charge transfer (MLCT)-type singlet and triplet transitions. The strong absorption band below 350 nm was assigned to the spin-allowed ^1^(π–π*) transition of the cyclometalated ligand, which was almost the same as the corresponding transition of the main ligand. The band between 350 nm and 400 nm was assigned to the spin-allowed metal-to-ligand charge transfer band (^1^MLCT and ^3^MLCT). 

The emission peaks of Ir-1 and Ir-2 in dichloromethane both appeared at 640 nm, indicating deep-red emission. Complexes Ir-1 and Ir-2 exhibited high luminescence efficiency, and the absolute phosphorescence quantum yields of their solutions were 0.64 and 0.55, respectively. Notably, phosphorescent iridium complexes with acetylacetone as an auxiliary ligand [30,35] have displayed an emission wavelength of 622 nm and an absolute quantum yield in the range of 0.2–0.32. In this study, the emission wavelengths of Ir-1 and Ir-2 with pyrone as an auxiliary ligand both underwent a red shift of about 20 nm, and the absolute phosphorescence quantum yield of Ir-1 was almost doubled. When using the same primary ligand, the pyrone auxiliary ligands in the phosphorescent iridium complexes Ir-1 and Ir-2 had the same O^O structure. However, compared with acetylacetone, the pyrone auxiliary ligand had more delocalized π electrons. A larger auxiliary ligand ma or ema increased the molecular volume of the corresponding Ir complexes, thereby increasing the distance between two adjacent emission centers. This directly decreased triplet–triplet annihilation (TTA).

The two complexes exhibited similar emission decay curves with excited-state lifetimes in the range of microseconds (Figure 2c), which means the emission originates from the triple excited state. The shorter the phosphor lifetime, the lesser the potential for triplet–triplet annihilation and triplet–polaron annihilation. The radiative decay rate constant (K_r_) of the two complexes was larger than the non-radiative decay rate (K_nr_), which may be due to the larger contribution of the MLCT state to the triple excited state T1, resulting in larger spin–orbit coupling and a larger K_r_. The relatively large K_r_ and short lifetime indicate that the triplet excitons can decay rapidly through the path of the radiative transition. This is important for manufacturing efficient OLED devices.

### 2.3. Theoretical Calculations

**Figure 3:**
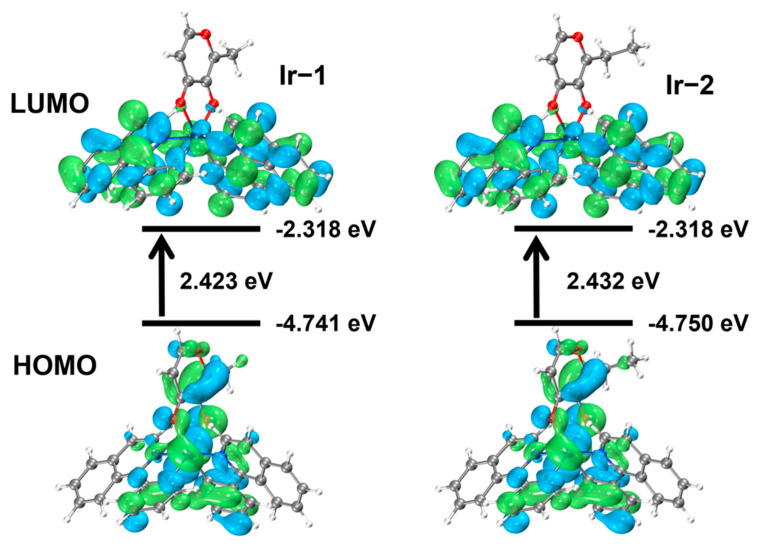
Distribution of calculated frontier molecular orbitals and HOMO–LUMO (H–L) energy gaps for Ir-1 and Ir-2.

To gain insight into the fundamental properties of Ir-1 and Ir-2, density functional theory (DFT) calculations were performed, and the optimized HOMO/LUMO distributions are shown in Figure 3. The results revealed that the lowest unoccupied molecular orbital (LUMO) of both complexes were concentrated on the metallic ligand and the central Ir atom. The high-density electron clouds on the central Ir atom suggested that the complexes underwent efficient metal-to-ligand charge transfer (MLCT), which was essential for obtaining a high PLQY. Changing the pyrone auxiliary ligand did not change the lowest unoccupied molecular orbital (LUMO) distribution of the complexes, as the LUMO energy levels of Ir-1 and Ir-2 were both −2.318 eV. The HOMO/LUMO distribution of the two complexes was significantly different from that of the LUMO distribution. Some orbitals were distributed in the quinoline region and on the central Ir atom of the metallacyclic ligand, while many others were distributed on the pyrone auxiliary ligand. This separation of these orbitals suggests that the compounds Ir-1 and Ir-2 have a bipolar transport ability, allowing electrons and holes to be transported through different channels. Additionally, a small portion of the electron cloud was distributed on the methyl group –CH_3_ of the substituent of the compounds Ir-1 and Ir-2 auxiliary ligand, whereas there was no electron cloud on the one –CH_2_– group, which was more abundant in Ir-2 than in Ir-1. This caused the electron cloud on –CH_3_ to be farther from the Ir atom, and thus, the HOMO energy level of Ir-2 was slightly lower than that of Ir-1. Energy gaps (E_g_) can be calculated according to the equation E_g_ = E_LUMO_ − E_HOMO_. Because the LUMO energy levels of the two compounds were identical and the HOMO energy level of Ir-1 was slightly higher than that of Ir-2, the E_g_ of Ir-1 (2.423 eV) should be slightly smaller than that of Ir-2 (2.432 eV). This narrower energy gap can facilitate charge injection, which is inherently more favorable to the emission of red light and is also why the PLQY of Ir-1 was slightly higher than that of Ir-2.

### 2.4. Electrochemical Properties 

**Figure 4:**
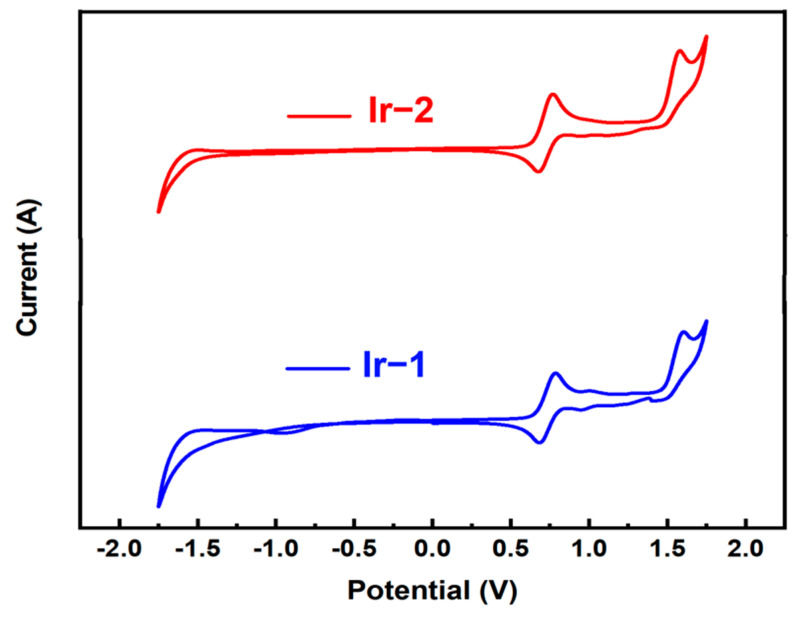
Cyclic voltammograms of complexes Ir-1 and Ir-2.

CV curves were obtained by dissolving Ir-1 and Ir-2 in dichloromethane, using ferrocene as the reference material, as shown in Figure 4. Only the oxidation curve was measured, and the curves underwent basically the same changes, with a reversible oxidation peak at 0.78 V, which was attributed to the redox couple of Ir^3+^/Ir^4+^ at the center of the complex. An irreversible oxidation peak appeared at about 1.60 V, which indicated that the different substituents of the two pyrone auxiliary ligands did not significantly affect the oxidation potential of either complex.

The HOMO energy level, the LUMO energy level, and the optical energy gap E_g_ of the two complexes were calculated according to the oxidation onset potential of the curve (Table 2). The oxidation onset potentials of Ir-1 and Ir-2 to ferrocene were calculated to be 0.50 eV and 0.46 eV, respectively. Based on the empirical formula of electrochemical calculations, $E_{HOMO}=-e(E_{onset}^{ox}+4.4)$ eV [36], the HOMO energy levels of complexes Ir-1 and Ir-2 were −4.90 and −4.86 eV, respectively. The E_g_ values of complexes Ir-1 and Ir-2 were calculated to be 2.15 eV and 2.05 eV from the absorption spectra (E_g_ = 1240/λ). Based on E_LUMO_ = E_g_ + E_HOMO_, the LUMO energy levels for complexes Ir-1 and Ir-2 were calculated to be −2.75 eV and −2.81 eV, respectively. The electrochemical calculations were in good agreement with the theoretical calculations of HOMO, LUMO, and E_g_, confirming the accuracy of the theoretical calculations. Compared with the reported (piq)_2_Ir(acac), which has a HOMO energy level of −5.48 eV, the HOMO energy levels of the two pyrone phosphorescent iridium complexes in this study were increased. The accompanying energy gap E_g_ was significantly reduced, which may red-shift the luminescence color, consistent with the photophysical performance results [35].

### 2.5. Electroluminescence Devices

**Figure 5:**
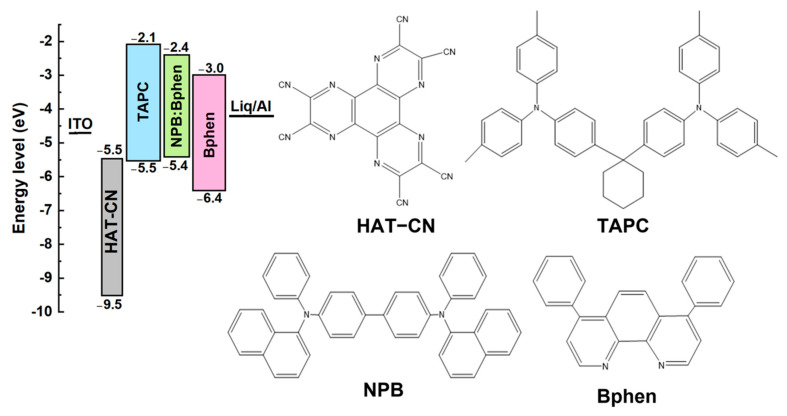
The structure of the EL and chemical structures of the materials used in PhOLEDs.

In order to understand the electrophosphorescence properties of the two red phosphors, six devices were fabricated using the same configuration: ITO/HAT-CN (30 nm)/TAPC (40 nm)/NPB:bphen:dopants (3, 6, 12%, 50 nm)/Lip (1 nm)/Al (100 nm). The devices with Ir-1 as doped emitters were denoted as D1-3, D1-6, and D1-12, while those with Ir-2 were denoted as D2-3, D2-6, and D2-12, respectively. The chemical structures of the materials used in the devices and their energy level diagrams are shown in Figure 5. N,N’-Di(1-naphthyl)-N,N’-diphenyl-(1,1′-biphenyl)-4,4′-diamine (NPB) and bathophenanthroline (bphen) were in a proportion of 1:1 and were the host material in the emitting layer. Hexaazatriphenylenehexacabonitrile (HAT-CN) was the hole-injecting layer (HIL). 1,1-Bis(4-methylphenyl)-aminophenyl-cyclohexane (TAPC) was the hole-transporting layer (HTL). Bphen was the electron-transporting layer (ETL). Indium tin oxide (ITO) was the anode. LiF/Al was the electron-injecting layer (EIL) and cathode. The energy levels of the NPB:bphen double-host material were close to the energy levels of the HTL TAPC and the electron transport material bphen. The device structure was reasonable, and there was almost no energy level barrier between adjacent interface layers, which helped reduce the turn-on voltage and improve the power conversion efficiency.

**Figure 6:**
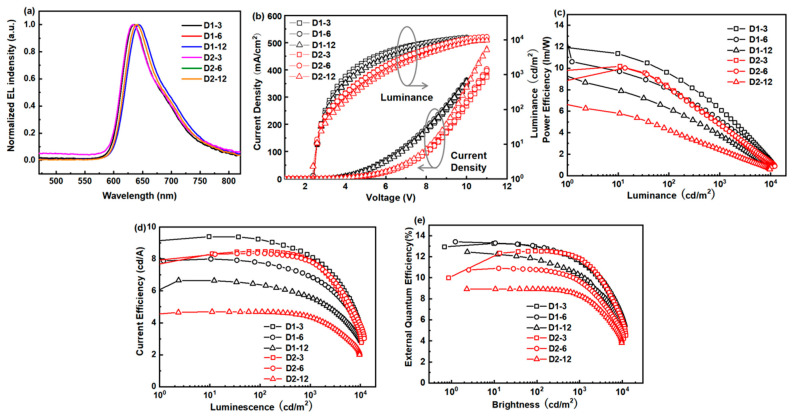
EL spectra (**a**), current density–voltage–luminance (J–V–L) characteristics (**b**), power efficiency curves (**c**), current efficiency curves (**d**), and external quantum efficiency curves (**e**) of devices D1-3, D1-6, D1-12, D2-3, D2-6, and D2-12.

Figure 6 and Table 4 show the luminescent performance of the six devices prepared using the complexes Ir-1 and Ir-2. As shown in Figure 6a, the EL and PL spectra of the complexes Ir-1 and Ir-2 were similar, suggesting that the luminescence of the Ir complexes originated from the radiative excitation of their triplet excitons. All devices exhibited deep-red emission, with emission wavelengths in the range of 633–644 nm. The EL spectra of the two compounds showed a peak at 600 nm, and the spectrum reached the maximum emission from less than 500 nm and extended to 800 nm. Because the human eye can more easily recognize light with shorter wavelengths than red light, the narrower left peak in the emission spectrum may help produce red with greater color purity [37,38,39]. The host did not display an emission peak in the EL spectra of any of the devices, indicating that energy from the host was completely transferred to the guest. These devices exhibited extremely low turn-on voltages (2.3–2.5 V), demonstrating that the device structure was rational. As shown in Figure 6b–e, the D1 device prepared with complex Ir-1 showed better luminescent performance than the D2 device prepared with complex Ir-2, which was consistent with the photophysical performance and theoretical calculations. Among these devices, D1-3 and D2-3 showed the best EL performance in their respective series of devices. When the doping concentration of complex Ir-1 was 3%, the turn-on voltage of D1-3 was 2.4 V; the maximum brightness was 11,140 cd∙m^−2^; and the maximum EQE, the maximum current efficiency, and the maximum lumen efficiency were 13.3%, 9.39 cd∙A^−1^, and 12.09 lm∙W^−1^, respectively. The emission peak appeared at 636 nm, with CIE chromaticity coordinates of (0.69, 0.31). When the doping concentration of complex Ir-2 was 3%, the turn-on voltage of D1-3 was 2.6 V; the maximum brightness was 11,218 cd∙m^−2^; and the maximum EQE, the maximum current efficiency, and the maximum lumen efficiency were 12.5%, 8.60 cd∙A^−1^, and 10.19 lm∙W^−1^, respectively. The emission peak appeared at 633 nm, with CIE chromaticity coordinates of (0.69, 0.31). Even when the brightness of D1-3 and D2-3 reached 1000 cd∙m^−2^, they still maintained EQE values of 11.6% and 12.0% and power efficiencies of 6.3 lm∙W^−1^ and 5.0 lm∙W^−1^, resulting in a small roll-off in device efficiency.

For the same complex, the luminescent performance decreased upon increasing the doping concentration, which resulted in a higher probability of triplet–triplet annihilation in the device. Even so, when the doping concentration reached 12%, D1-12 and D2-12 still maintained a maximum EQE of 12.4% and 9.2%, and the maximum brightness remained above 1000 cd m^−2^. This was attributed to the excellent double-carrier transport capacity of compounds Ir-1 and Ir-2, which minimized the impact of triplet–triplet annihilation on device performance caused by a high doping concentration. This may also help reduce the efficiency roll-off in OLEDs and achieve devices with high repeatability, thus reducing the difficulty in device fabrication and facilitating future large-scale industrial production.

## 3. Materials and Methods

### 3.1. General Information

All chemicals and reagents were purchased from commercial sources and used without further purification. ^1^H NMR spectra were recorded using a 400 MHz BrukerDRX-500 spectrophotometer in CDCl_3_ at room temperature. Mass spectra were obtained on an ATI-QSTAR MS spectrometer operating in FAB+ mode. Thermogravimetric analysis (TGA) and differential scanning calorimetry (DSC) were performed on a Netzsch STA 449F instrument under nitrogen flow at a heating rate of 31 °C min^−1^. Single crystals of the two iridium complexes were kept in a dark location. Ir-1 crystals were grown in acetonitrile and DMSO, and Ir-2 crystals were grown in the dark in acetonitrile, methyl alcohol, and tetrahydrofuran. Single-crystal X-ray diffraction (XRD) patterns were collected at 193 K on a Bruker D8 Venture diffractometer with graphite monochromated Cu-Kα radiation. UV–VIS absorption and fluorescence spectra were measured on the Edinburgh Instruments FS5 Spectrofluorometer. Cyclic voltammograms (CVs) were measured using a conventional three-electrode configuration and an electrochemical workstation (PG STAT302) at a range of −2~2 V with a platinum working electrode, in which the onset oxidation and reduction potentials relative to Fc/Fc^+^(0.69 V) were represented. The LUMO level was calculated. The geometries of Ir-1 and Ir-2 in the ground state were optimized with dispersion-corrected density functional theory (DFT-D3) at the PBE0-D3/def2-SVP level. The IEFPCM implicit solvent model was used in all calculations. To obtain the electron structure with greater accuracy, single-point calculations were performed for these optimized structures with the TPSSh functional and the def2-TZVP basis set. All calculations were performed using the Gaussian 16 program. The frontier molecular orbitals were visualized using the Visual Molecular Dynamics (VMD) program [40].

### 3.2. Device Fabrication and Measurement

Patterned indium tin oxide (ITO)-coated glass substrates (15 Ω square-1) were cleaned successively with deionized water, detergent solution, acetone, and methanol in an ultrasonic bath and then exposed to UV ozone for 15 min. After the substrates were transferred to a vacuum chamber, devices were fabricated by sequentially depositing organic layers in one run under high vacuum (<3 × 10^−5^ Pa) thermal evaporation onto a precleaned indium tin oxide–glass substrate. The luminance and electroluminescence (EL) spectra were recorded by Minolta LS-110 Luminance meter and Ocean Optics USB-4000 spectrometer, respectively. The current density–voltage characteristics were measured using an HP4140B picoammeter. The EQE was calculated from the EL spectrum, and luminance and current density were calculated assuming a Lambertian emission distribution. Luminance and EL spectra were recorded with a Minolta LS-110. Temperature-dependent EL spectra were measured using a liquid-nitrogen-cooled optical cryostat (Optistat DNV, Oxford Instruments) with an ITC503S temperature controller. Transient EL spectra were measured using a Keysight DSO1012A oscilloscope equipped with a LINI-UTP3313TFL-11-regulated DC power supply and a VICTOR DDS signal generator counter. PhOLEDs were fabricated using the following optimized device configuration: ITO/HAT-CN (3 nm)/TAPC (40 nm)/NPB:bphen:dopants (3%, 6%, and 9%, 10 nm)/bphen (50 nm)/Liq (1 nm)/Al (100 nm).

### 3.3. Compound Synthesis

Synthesis of (piq)_2_Ir_2_Cl_2_(piq)_2_. Cyclometalated iridium dimers (Ir(C^N)_2_(μ-Cl))_2_ (C^N) = 1-phenylisoquinoline (piq) were prepared according to a modified Nonoyama procedure [41]. IrCl_3_∙3H_2_O (15.57 g, 43.7 mmol), 1-phenylisoquinoline (piq) (96.2 mmol, 2.2 equiv.) and a solvent mixture of 120 mL of 2-methoxyethanol/water (3:1, *v*/*v*) was added to a 500 mL two-neck flask and heated to reflux at 120 °C for 18 h under a nitrogen atmosphere. Water (45 mL) was added to the reaction mixture after the mixture was cooled to room temperature. The red precipitate was collected by filtration and washed using acetone (30 mL), deionized water (40 mL × 2), and acetone (30 mL × 2), successively. Finally, the wet solid was completely dried to obtain the chloride–bridged dimer complex. The isolated yield was between 50% and 70%.

Synthesis of Ir(piq)_2_(ma) (Ir-1) and Ir(piq)_2_(ema) (Ir-2). Without further purification, a mixture of the red powder dimer (1.6 mmol), Na_2_CO_3_ (9 mmol), and maltol or ethyl maltol (8 mmol) was added to 2-methoxyethanol (65 mL). The reaction mixture was stirred at 135 °C under an argon atmosphere for another 12 h. After cooling to room temperature, the reaction mixture was filtered and evaporated to remove the solvent. The crude product was washed with ethanol three times and dried to obtain the target complexes Ir-1 and Ir-2. The isolated yield was between 30% and 50%.

Ir-1: dark-red powder, yield 35%. 1H NMR (400 MHz, chloroform-d) δ 9.00–8.94 (m, 2H), 8.74 (d, J = 6.4 Hz, 1H), 8.43 (d, J = 6.4 Hz, 1H), 8.21 (d, J = 8.0 Hz, 2H), 7.90 (dd, J = 5.8, 3.7 Hz, 2H), 7.75–7.65 (m, 4H), 7.61 (d, J = 5.1 Hz, 1H), 7.46 (dd, J = 13.1, 6.3 Hz, 2H), 6.92–6.85 (m, 2H), 6.69–6.62 (m, 2H), 6.49 (d, J = 5.1 Hz, 1H), and 6.37–6.30 (m, 2H), 2.40 (s, 3H). MALDI-TOF-MS (*m*/*z*): calcd. for C36H25IrN2O3: 726.1573; found: 727.1577 [M+1]+. Anal. calcd for C_36_H_25_IrN_2_O_3_: C, 59.55; H, 3.47; N, 3.86. Found: C, 59.46; H, 3.60; N, 3.85.

Ir-2: dark-red powder, yield 46%. 1H NMR (400 MHz, chloroform-d) δ 8.98 (dt, J = 6.8, 3.1 Hz, 2H), 8.74 (d, J = 6.4 Hz, 1H), 8.44 (d, J = 6.4 Hz, 1H), 8.21 (d, J = 8.0 Hz, 2H), 7.90 (dd, J = 4.7, 3.0 Hz, 2H), 7.75–7.66 (m, 4H), 7.64 (d, J = 5.1 Hz, 1H), 7.45 (dd, J = 10.6, 6.4 Hz, 2H), 6.93–6.85 (m, 2H), 6.70–6.62 (m, 2H), 6.49 (d, J = 5.1 Hz, 1H), 6.35 (td, J = 7.6, 1.1 Hz, 2H), 2.91 (dd, J = 15.2, 7.6 Hz, 1H), 2.73 (dd, J = 15.1, 7.5 Hz, 1H), and 1.12 (t, J = 7.5 Hz, 3H). MALDI-TOF-MS (*m*/*z*): calcd. for C37H27IrN2O3: 740.1729; found: 741.1716 [M+1]+. Anal. calcd for C_37_H_27_IrN_2_O_3_: C, 60.04; H, 3.68; N, 3.78. Found: C, 59.96; H, 3.81; N, 3.78.

## 4. Conclusions

Most previously reported iridium phosphors with 1-phenylisoquinoline as the cyclometalating ligand show yellow-to-orange phosphorescence with maximum wavelengths below 630 nm. The auxiliary ligands are always β-diketones, such as acetylacetone. In this work, two novel iridium complexes were developed for the first time using 1-phenylisoquinoline as the cyclometalating ligand and pyrone as an auxiliary ligand to adjust the light-emitting properties. The auxiliary ligand obviously affected the excited-state energy and emission colors of the iridium complexes. The PL and EL quantum yields were also influenced. The pyrone-type auxiliary ligand increased the HOMO of the iridium complexes, resulting in a decrease in the excited-state energies and a bathochromic shift in their phosphorescence peaks relative to the complex with an acetylacetone auxiliary ligand. The phosphorescence of the complexes with the pyrone auxiliary ligand produced a deep-red color at 640 nm in its PL spectrum and produced deep-red EL with emission peaks at 633–644 nm and CIE coordinates of (0.70, 0.33) in an OLED. The Ir(piq)_2_(ma) complex showed even better performance, but it did not show a high PLQY due to the presence of –CH_3_ groups. However, the carrier-transporting property was improved and the triplet–triplet annihilation was reduced, which improved the EL performance of the corresponding iridium complexes in OLEDs. The iridium phosphor containing maltol exhibited an external quantum efficiency of 13.4% in the doped PhOLED. This is the first report of the use of maltol and ethyl maltol as auxiliary ligands in iridium phosphors, providing a practical molecular design strategy to produce saturated red iridium phosphors using pyrone as an auxiliary ligand. This is a special kind of doping technology, and it should as such have a more general use in emitter design.

## Figures and Tables

**Table 1:** Crystal data of Ir-1 and Ir-2.

Complex	Ir-1	Ir-2
Formula	C_36_H_25_IrN_2_O_3_	C_37_H_27_IrN_2_O_3_
Formula weight	725.78	739.80
Crystal system	Monoclinic	Monoclinic
Space group	C2/c	P2_1_/c
a (Å)	11.4553 (5)	10.0394 (4)
b (Å)	15.4613 (6)	21.4133 (8)
c (Å)	20.2487 (7)	14.3292 (6)
α (⁰)	90	90
β (⁰)	101.138 (2)	104.506 (2)
γ (⁰)	90	90
*V* (Å^3^)	3518.78 (20)	2982.2 (2)
*Z*	4	4
*ρ*_calc_ (g∙ cm^−3^)	1.370	1.648
Reflections collected	11,912	24,146
Independent reflections	3228	5855
RF, Rw (F2) (all data)	0.05	0.0485
RF, Rw (F2) (I > 2σ(I))	0.0426	0.0436
GOF	1.108	1.187

**Table 2:** Coordination bonding parameters (Å, °) of Ir-1 and Ir-2 complexes.

Complex	Ir–C	Ir–N	Ir–O	C–Ir–C	N–Ir–N	O–Ir–O	C–Ir–O	N–Ir–O	C–Ir–N
Ir-1	1.971 (7)	2.040 (5) 2.040 (5)	2.145 (5)	87.4 (4)	175.6 (2)	78.4 (3)	174.60 (16)	96.52 (17)	80.0 (2)
	1.972 (7)		2.145 (5)				174.60 (16)	96.52 (17)	80.0 (2)
							97.2 (3)	86.93 (17)	96.7 (2)
							97.2 (3)	86.93 (17)	96.7 (2)
Ir-2	1.971 (6)	2.022 (5)	2.173 (4) 2.155 (4)	89.4 (2)	175.8 (2)	78.3 (2)	169.61 (19)	92.44 (17)	80.5 (2)
	1.981 (5)	2.030 (5)					175.4 (2)	86.62 (18)	96.5 (2)
							93.5 (2)	90.87 (18)	97.4 (2)
							99.14 (19)	96.62 (18)	79.5 (2)

**Table 3:** Photophysical data of Ir-1 and Ir-2.

Complex	*λ*_abs_^a^ (nm)	*Λ*_em_^a^ (nm)	HOMO/LUMO ^b^ (eV)	E_g_^c^ (eV)	Φ_p_^d^ (%)	Τ ^a^ (μs)	K_r_^e^ (×10^5^s^−1^)	K_nr_^e^ (×10^5^s^−1^)
Ir-1	229, 270, 346, 397	640	−4.90/−2.75	2.15	0.64	0.13	4.92	2.77
Ir-2	232, 273, 345, 398	640	−4.86/−2.18	2.05	0.55	0.13	4.23	3.46

^a^ Measured in CH_2_Cl_2_ at a concentration of 1.0 × 10^−5^ mol L^−1^ at room temperature. ^b^ Calculated from the empirical equation HOMO = -e($E_{onset}^{ox}$ + 4.4) eV and LUMO = E_g_ + E_HOMO_. ^c^ Calculated from the empirical equation E_g_ = 1240/λ. ^d^ Φ_p_ emission quantum yields were measured with a CH_2_Cl_2_ standard. ^e^ Radiative decay rate K_r_ = Φ_p_/τ, and non-radiative decay rate Knr = (1 − Φ_p_)/τ.

**Table 4:** EL characteristics of red PhOLEDs.

Devic	Maximum Brightness (cd∙m^−2^)	Current Efficiency (cd∙A^−1^)	Lumen Efficiency (lm∙W^−1^)	Maximum EQE (%)	Maximum Emission Wavelength (nm)	CIEx	CIEy
D1-3	11,140	9.39	12.09	13.3	636	0.69	0.31
D1-6	11,660	8.10	10.65	13.4	641	0.69	0.30
D1-12	10,130	6.74	8.98	12.4	645	0.70	0.30
D2-3	11,218	8.60	10.19	12.5	633	0.69	0.31
D2-6	13,298	8.37	10.05	11.1	640	0.69	0.30
D2-12	10,653	4.70	6.61	9.2	641	0.70	0.30

## Data Availability

The data presented in this study are contained within the article.
